# Low-Molecular-Weight Organogelators Based on *N*-dodecanoyl-L-amino Acids—Energy Frameworks and Supramolecular Synthons

**DOI:** 10.3390/ma16020702

**Published:** 2023-01-11

**Authors:** Barbara Miroslaw, Oleg M. Demchuk, Roman Luboradzki, Katarzyna Tyszczuk-Rotko

**Affiliations:** 1 Institute of Chemical Sciences, Faculty of Chemistry, Maria Curie-Sklodowska University in Lublin, 20-031 Lublin, Poland; 2 Faculty of Medicine, The John Paul II Catholic University of Lublin, 1h-Konstantynów St., 20-708 Lublin, Poland; 3 Institute of Physical Chemistry, Polish Academy of Sciences, 01-224 Warsaw, Poland

**Keywords:** organogels, drug delivery, energy frameworks, supramolecular synthons, amides, self-assembled fibrillary network, SAFiN

## Abstract

Lauric acid was used to synthesize the low-molecular-weight organogelators (LMOGs), derivatives of two endogenous (L)-alanine, (L)-leucine, and three exogenous (L)-valine, (L)-phenylalanine, and (L)-proline amino acids. The nature of processes responsible for the gel formation both in polar and in apolar solvents of such compounds is still under investigation. Knowing that the organization of surfactant molecules affects the properties of nano scale materials and gels, we decided to elucidate this problem using crystallographic diffraction and energy frameworks analysis. The single crystals of the mentioned compounds were produced successfully from heptane/^t^BuOMe mixture. The compounds form lamellar self-assemblies in crystals. The energetic landscapes of single crystals of a series of studied amphiphilic gelators have been analyzed to explore the gelling properties. The presented results may be used as model systems to understand which supramolecular interactions observed in the solid state and what energy contributions are desired in the designing of new low-molecular-weight organic gelators.

## 1. Introduction

Pharmaceutical gels are willingly applied in pharmaceutical formulations where active pharmaceutical ingredients (API) are combined to yield a final medicinal product of well-defined properties. The stability of the obtained formulation is a crucial issue in the pharmaceutical and cosmetics industries [1]. Stability, in the case of pharmaceutical products, is an ability to maintain the original form and properties (to ensure that the product maintains its intended quality, safety and efficacy as well as functionality) under the influence of various factors, such as time, humidity, temperature, etc. when stored under appropriate conditions throughout the shelf life [2]. The problem of stability is especially visible in the cases of semiliquid formulations, gels, and nanoparticle-based drugs [3,4,5,6]. The latter offer the unique possibility to overcome cellular barriers in order to improve the delivery of active ingredients. Such a gel form may improve a medicine’s efficacy and decrease side effects. Moreover, the drug distribution to target sites via passive and active targeting may be modulated [7,8,9]. A hydrophilic nanoparticle can be an effective carrier for insoluble drugs of low bioavailability [10]. The properties of the nanoparticle-based drugs depend on the particle size. On the one hand, the small particles have an extremely large specific surface, and therefore large specific API capacity. On the another hand, the size of nanoparticles modifies the ability of the drug to pass from the blood vessels to the targeted site destination [11,12]. It helps to reach the extracellular fluid of the tissue by passing through the blood capillary wall, next going by untargeted cells, and approaching the target cells [13]. The shape is also one of the crucial parameters that strongly influences the bioactivity of the nanoparticles [14]. 

Small particles are unstable with respect to agglomeration to the bulk caused by van der Waals forces, in the absence of any repulsive forces. This repulsive interaction can be facilitated by two methods: electrostatic and steric stabilization [15]. Electrostatic stabilization is achieved by creation of a surface charge [16], while steric stabilization by attaching the polymers and surfactants at the surface of the particles [17].

In many cases, the shape [18], size, as well as the surface charge and coating are created during the initial process of the nanoparticle formation where the bottom up synthesis of nanoparticles is realized in the presence of surfactants [15,19,20]. At low concentrations in solution, surfactants exist as individual molecules. At higher concentrations, the hydrophobic interactions cause minimization of the number of contacts between nonpolar surfactant tails and water [21]. They undergo a spontaneous self-assembly to form thermodynamically stable aggregates the parameters which depend on surfactant structure, concentration, solution composition, and general conditions. 

Another pharmaceutical application of surfactants leads in their ability to form organogels [22]. The organogelators may gel an injectable oil solutions of API and to sustain its release in very specific manner [23]. The gelation process depends on the interaction forces between the organogelator molecules, which at a specific concentration, forms a 3D net of nano fibers, constructed by hydrogen bonds, London dispersion forces, and electrostatic interactions that immobilizes solvent molecules [24]. There are numbers of organogels described in the literature [25,26], but relatively few have been proposed for pharmaceutical usage mostly because of information on their usual bioincompatibility and toxicity [22]. 

In both nanoparticle and organogel drug delivery systems, the biocompatibility of the surfactants used is a crucial issue. Herein, we decided to overcome the natural toxicity of the common surfactants by condensation of naturally occurred molecules: fatty (lauric) acid and amino acids. Indeed, several studies already showed that such amides can act as efficient and biodegradable gelators [22,27,28,29,30].

The role of the interactions between such surfactants during gelation is still under investigation. The self-assembled fibrillary network (SAFiN) formation was proved to be related to the hydrogen bond’s formation ability [31,32,33,34,35]. Obviously there is no direct possibility to process the mapping of interaction in the nano fibers or on nano particle surface, but a good approximation to those interactions may provide crystal diffraction studies [36]. The organization of surfactant molecules affects the properties of nano materials and organogels. It depends highly on the interactions between the surfactant molecules in terms of the geometry as well as the energetic contributions. Therefore, we decided for the first time to apply the energetic landscapes approach proposed by Mackenzie et al. [37] to explore the gelling abilities of selected low-molecular-weight organogelators (LMOGs)—*N*-dodecanoyl-L-amino acid derivatives. The energy framework approach was previously successfully applied in explaining many problems regarding polymorphism, co-crystals, supramolecular synthons, solid state reactions and sol-to-gel transition process [38,39,40,41,42,43,44]. 

Amides based on amino acids linked to long alkyl chains (>C4) do not crystallize easily to give single crystals of good quality. In the Cambridge Structural Database (version 5.42 with updates) [45] there are only seven entries of such crystals including one hydrate, and two cocrystals [22,30,46,47]. We succeeded in obtaining single crystals suitable for X-ray diffraction analysis for the whole series of the studied compounds. The crystal structure of 3-Phe was published before (CSD code WUWJUW) [30]; however, its crystal packing was not analyzed in detail nor were the energy landscapes calculated. 

Here, we present the study of crystal packing and energy frameworks of a series of amphiphilic gelators based on *N*-dodecanoyl-L-amino acid derivatives for which we managed to obtain single crystals by recrystallization from heptane/^t^BuOMe mixture to explore if the solid state supramolecular synthons and the energy frameworks may be an indicator for desirable gelling properties. Obtained gelators were proved to be suitable for organogels and hydrogels formation.

## 2. Materials and Methods

### 2.1. General Chemistry

Melting points were recorded in open capillaries and are uncorrected. ^1^H NMR (500 MHz) and ^13^C NMR (125 MHz) spectra were taken on a Bruker AVANCE 500 MHz spectrometer using CDCl_3_ as a solvent (Figures S1–S5). 

Compounds **1–5** were prepared according to our previously reported protocols [48] and their spectral and other physicochemical properties were consistent with the literature data [29,49].

### 2.2. Preparation of the Gels

**Figure 1:**
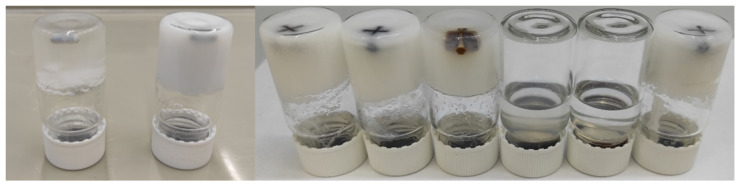
Gelation behavior of 1-Ala in various solvents in order from left: in toluene (transparent gel), water, t-BuOMe, petroleum ether (120–140 °C), CHCl_3_ (opaque gels), no gelation in alcohols C_2_H_5_OH and n-C_5_H_11_OH, and finally a gel in a mixture of water/ethyl alcohol (6/4 *v*/*v*).

The dry glass vial equipped with the stirrer magnet was charged with 10 mL of proper solvent and 1 g of 1-Ala. The vial was closed, and the reaction mixture was heated in an oil bath at 20 °C above the boiling point of the solvent used, maintaining continuous stirring for 5 min. Next the vial was cooled down in an ice bath without the stirring to 5–0 °C. After the cooling was completed the stable at ambient (and lower) temperature gels in toluene, water, t-BuOMe and petroleum ether were formed as presented in Figure 1. The gel obtained with CHCl_3_ melts above 19 °C. The gelation in alcohols, DMF, DMSO was unsuccessful; however, gel obtained with 40% *v*/*v* aqueous ethyl alcohol was stable for a few days at ambient temperature.

### 2.3. X-ray Diffraction

The crystal structures of the analyzed compounds 1-Ala, 2-Pro, 3-Phe, 4-Leu, and 5-Val were determined based on the X-ray diffraction experiment performed on single crystals at 120 K on SuperNova X-ray diffractometer, CuK*α* radiation (*λ* = 1.54184 Å). The structural models were obtained by direct methods using the SHELXS-97 program [50] and refined by the full-matrix least-squares method on *F*^2^ using the SHELXL-97 program implemented on Olex2.refine package [51]. Crystal structure of 3-Phe was redetermined. The first report was published by Chen et al. with final *R*_1_ = 10.96% [30]. The propan-2-yl fragment of the valine derivative in 5-Val was refined as disordered over two positions with site occupation factors being of 0.72 and 0.28. The compounds crystallized in non-centrosymmetric space groups. The absolute configuration was established by reference to the unchanging chiral center in the synthetic route because the Flack test results were ambiguous (no elements heavier than O in the crystal structure). 

Crystal Data for 1-Ala, C_15_H_29_NO_3_ (*M* = 271.39 g/mol): triclinic, space group *P*1 (no. 1), *a* = 4.8852(2) Å, *b* = 5.5958(2) Å, *c* = 30.2891(13) Å, *α* = 90.765(3), *β* = 91.034(4), *γ* = 105.561(4), *V* = 797.37(6) Å^3^, *Z* = 2, *T* = 120.10(10) K, μ(CuKα) = 0.615 mm^−1^, *Dcalc* = 1.1303 g/cm^3^, 15,118 reflections measured (5.84 ≤ 2Θ ≤ 144.06), 5297 unique (*R*_int_ = 0.0340, R_sigma_ = 0.0330) which were used in all calculations. The final *R*_1_ was 0.0448 (I ≥ 2σ(I)) and *wR*_2_ was 0.1537 (all data). CCDC No. 2226607.

Crystal Data for 2-Pro, C_17_H_31_NO_3_ (*M* = 297.43 g/mol): monoclinic, space group *P*2_1_ (no. 4), *a* = 6.65959(13) Å, *b* = 8.08116(17) Å, *c* = 15.9905(3) Å, *β* = 90.4021(17)°, *V* = 860.54(3) Å^3^, *Z* = 2, *T* = 120.10(10) K, μ(CuKα) = 0.612 mm^−1^, *Dcalc* = 1.1478 g/cm^3^, 4825 reflections measured (5.52° ≤ 2Θ ≤ 143.54°), 2282 unique (*R*_int_ = 0.0146, R_sigma_ = 0.0165) which were used in all calculations. The final *R*_1_ was 0.0297 (I ≥ 2σ(I)) and *wR*_2_ was 0.0900 (all data). CCDC No. 2226609.

Crystal Data for 3-Phe, C_21_H_33_NO_3_ (*M* = 347.48 g/mol): orthorhombic, space group P2_1_2_1_2_1_ (no. 19), *a* = 5.3354(2) Å, *b* = 12.7541(6) Å, *c* = 28.9824(14) Å, *V* = 1972.19(16) Å^3^, *Z* = 4, *T* = 120.10(10) K, μ(CuKα) = 0.607 mm^−1^, *Dcalc* = 1.1703 g/cm^3^, 5142 reflections measured (6.1° ≤ 2Θ ≤ 140.02°), 3303 unique (*R*_int_ = 0.0432, R_sigma_ = 0.0541) which were used in all calculations. The final *R*_1_ was 0.0487 (I ≥ 2σ(I)) and *wR*_2_ was 0.1494 (all data). CCDC No. 2231565—redetermination of CCDC No. 1959468 CSD Refcode WUWJUW [30].

Crystal Data for 4-Leu, C_18_H_35_NO_3_ (*M* = 313.47 g/mol): orthorhombic, space group *P*22_1_2_1_ (no. 18), *a* = 5.32892(18) Å, *b* = 13.4149(4) Å, *c* = 27.0501(8) Å, *V* = 1933.73(10) Å^3^, *Z* = 4, *T* = 120.10(10) K, μ(CuKα) = 0.564 mm^−1^, *Dcalc* = 1.0767 g/cm^3^, 6310 reflections measured (6.54° ≤ 2Θ ≤ 144.2°), 3157 unique (*R*_int_ = 0.0240, R_sigma_ = 0.0220) which were used in all calculations. The final *R*_1_ was 0.0387 (I ≥ 2σ(I)) and *wR*_2_ was 0.1046 (all data). CCDC No. 2226608.

Crystal Data for 5-Val, C_17_H_33_NO_3_ (*M* = 299.44 g/mol): orthorhombic, space group *P*2_1_2_1_2_1_ (no. 19), *a* = 32.7663(7) Å, *b* = 9.9958(2) Å, *c* = 5.68440(11) Å, *V* = 1861.79(7) Å^3^, *Z* = 4, *T* = 120.10(10) K, μ(CuKα) = 0.566 mm^−1^, *Dcalc* = 1.0677 g/cm^3^, 8144 reflections measured (9.24° ≤ 2Θ ≤ 143.98°), 3259 unique (*R*_int_ = 0.0275, R_sigma_ = 0.0228) which were used in all calculations. The final *R*_1_ was 0.0639 (I ≥ 2σ(I)) and *wR*_2_ was 0.2052 (all data). CCDC No. 2226610.

CCDC No. 2226607–2226610 and 2231565 contain the supplementary crystallographic data for this paper. These data can be obtained free of charge via http://www.ccdc.cam.ac.uk/conts/retrieving.html (or from the Cambridge Crystallographic Data Centre, 12, Union Road, Cambridge CB2 1EZ, UK; fax: +44-1223-336033) (accessed on 13 December 2022).

### 2.4. Energy Framework Calculations

Hirshfeld analysis is a well-known method for exploring crystal data to gain deeper insight into the characteristics of intermolecular interactions in a solid state [52,53,54,55]. For each crystal, a cluster of the closest molecules was generated and the unique pair energies have been calculated using CrystalExplorer [56,57,58] with the CE-B3LYP model derived from wavefunctions computed by Gaussian09 [59]. The energies were scaled by factors 1.057, 0.74, 0.871, and 0.618 for coulombic, polarization, dispersion and repulsion energy terms, respectively, according to Mackenzie et al. [37]. The initial geometry for the calculations was taken from the single crystal diffraction experiments. In the case of 1-Ala with 2 independent molecules in the unit cell, the lattice energy is given as an average of the lattice sums for each of them. In 5-Val, the two disordered orientations of the molecule with the partial occupancies were included in calculations 

## 3. Results

**Scheme 1:**
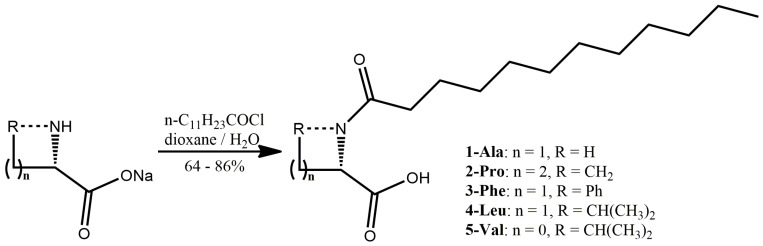
Molecular structure of compounds 1-Ala, 2-Pro, 3-Phe, 4-Leu, and 5-Val.

The synthesis of the five *N*-dodecanoyl-L-amino acid derivatives was based on the optimization of well-defined approaches which usually allow obtaining the amino acids of high purity in acceptable yields [48]. Thus, the lauric acid was converted into the lauroyl chloride in the reaction with thionyl chloride (Scheme 1). The synthesis was carried out in toluene solution and the obtained product has the satisfied quality to be used without additional purification. Such obtained lauroyl chloride was subjected the Schotten-Baumann with in situ prepared sodium salts of five selected natural amino acids: endogenous sodium (L)-alaninate, (L)-leucinate, and exogenous (L)-valinate, (L)-phenylalaninate, and (L)-prolinate (Scheme 1). The syntheses were carried out in 50% water—dioxane solution solubilizing the substrates and products, while the acidifying the postreaction mixture with aqueous hydrochloric acid effected in separation of mineral phase containing the majority of impurities. The additional purification of the desirable products of high purity was achieved by a single crystallization from heptane/^t^BuOMe mixture. The alternative approaches tested to prepare the targeted fatty acid amides were less efficient [27,29,60,61]. 

The enriched with gold nanoparticles *N-*lauroyl-L-alanine (1-Ala)-based hydrogel was tested as a supramolecular drug carrier [62]. The gelation properties of 1-Ala, 3-Phe, 4-Leu, and 5-Val were reported by MacLachlan and Hum et al. in the inversion test [30]. The example of gelation by 1-Ala is presented in Figure 1. The studied compounds exhibit the ability to form gels both in polar and in apolar solvents. In toluene (apolar) the gel is transparent, whereas in water (polar) it is opaque. Other tests were performed on 9% gels of 1-Ala in different solvents: the gels obtained with t-BuOMe and petroleum ether were stable at ambient temperature. The gel obtained with CHCl_3_ melts above 19 °C. The gelation of alcohols was not successful; however, gel obtained with 40% *v*/*v* aqueous ethyl alcohol was stable for a few days at ambient temperature, and it changed slowly from nanofiber (gel) to a crystalline form by dissolving-crystallisation process. The last observation proved that gels could be obtained in solvent mixtures, where the solubility of the gelator in at least one of the solvents used is low. The stability of gels is higher in the cases, where a possibility of dissolving of nanofiber of gelators and its recrystallization is limited. On the other hand, solvents in which the gelator is well soluble (CHCl_3_, EtOH, DMF, DMSO) might not be gelated in a pure form.

### Crystal Structure and Energetic Landscape Analysis

As pure enantiomers all studied compounds crystallized in chiral space groups (*P*1, *P*2_1_, *P*2_1_2_1_2_1_ and *P*22_1_2_1_, Table S1)-Ala has two symmetrically independent molecules in the unit cell-Val exhibited positional disorder of the propan-2-yl moiety. The heteroatoms formed bonds within the expected range of bond lengths (Table S2). 

**Figure 2:**
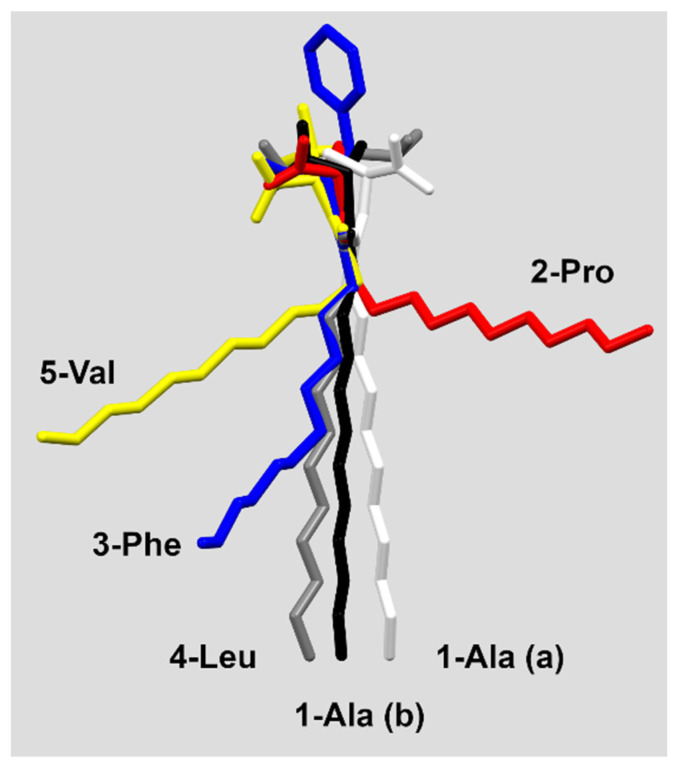
Conformation differences in the studied molecules.

The conformation of the molecules (especially the alkyl chain) seems not to determine the crystal packing mode. The 3-Phe, 4-Leu and 5-Val which form similar folded layers show the conformations of the aliphatic chain from synclinal (5-Val) through anticlinal (3-Phe) up to antiperiplanar (4-Leu) ones (Figure 1 and Figure 2, Table S2). 

**Figure 3:**
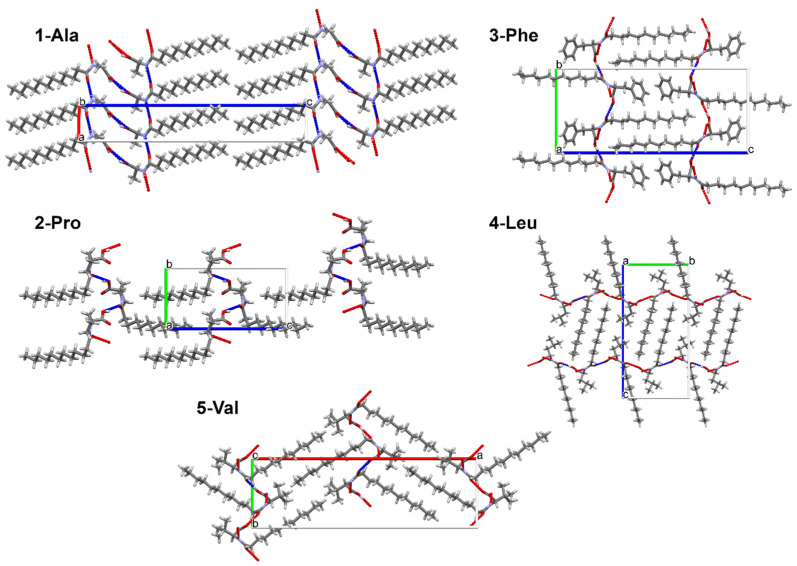
Crystal packing in structures of 1-Ala, 2-Pro, 3-Phe, 4-Leu, 5-Val.

The separation of hydrophilic and lipophilic regions (Figure 3) which may favor the gelation process occurs in a special way. The connection between gelling ability of some amino acid surfactants and 2-dimensional packing type was considered previously by Bastiat and Leroux [22]. To study such potential relationships, in the crystal packing analysis we decided to complement the study by the energy framework calculations.

Table 1 contains the pairwise interaction energies for the analyzed crystals calculated using the CrystalExplorer at CE-B3LYP/6-31G(d,p) level in kJ/mol. All of the values were scaled by factors 1.057, 0.74, 0.871, and 0.618 for coulombic, polarization, dispersion, and repulsion energy terms, respectively, according to Mackenzie et al. [37].

**Figure 4:**
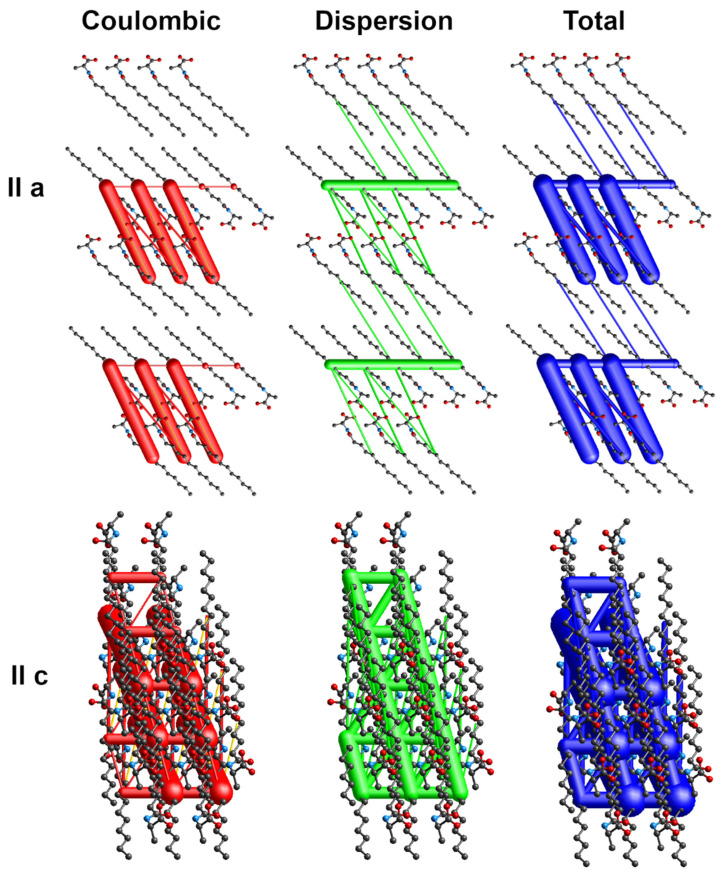
Energy frameworks for 1-Ala. View along *a* and *c* directions. Various colors represent various energy terms: red—coulombic, yellow—polarization, green—dispersion, and blue—total energy. The applied scale for tube size 150, the energy cut-off 5 kJ/mol.

**Figure 5:**
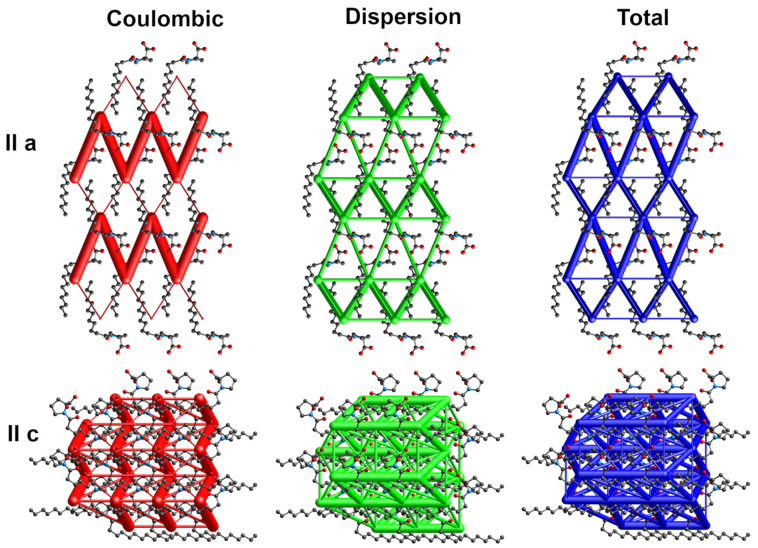
Energy frameworks for 2-Pro. View along *a* and *c* directions. Various colors represent various energy terms: red—coulombic, yellow—polarization, green—dispersion, and blue—total energy. The applied scale for tube size 150, the energy cut-off 5 kJ/mol.

**Figure 6:**
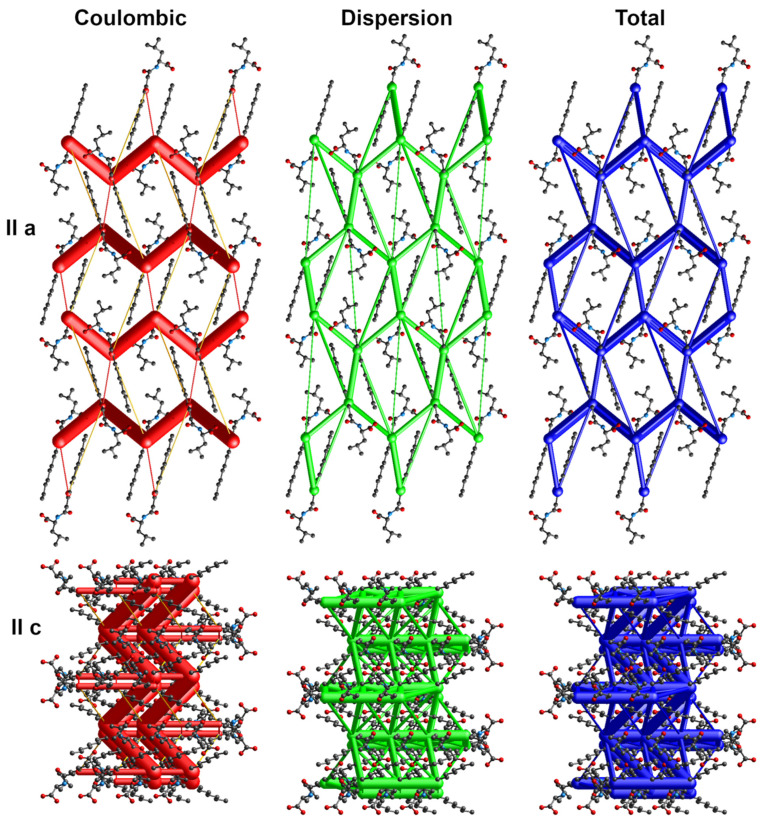
Energy frameworks for 4-Leu. View along *a* and *c* directions. Various colors represent various energy terms: red—coulombic, yellow—polarization, green—dispersion, and blue—total energy. The applied scale for tube size 150, the energy cut-off 5 kJ/mol.

The energy frameworks indicate the dominance of coulombic and dispersion energies in the crystal nets. Figure 4, Figure 5 and Figure 6 show selected energy frameworks viewed along *a* and *c* directions. Various colors represent various energy terms: red—coulombic, yellow—polarization, green—dispersion, and blue—total energy. The scale for tube size was 150, the energy cut-off 5 kJ/mol. 

**Figure 7:**
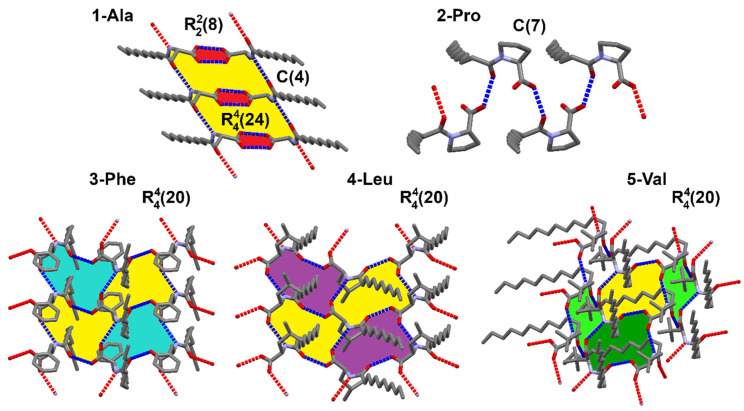
Supramolecular motifs according to graph set synthons notation created by strong hydrogen bonds found in the studied crystals.

In crystal packing, there is an evident division for hydrophilic and lipophilic sections (Figure 3). Within the hydrophilic regions the amide and carboxylic groups participate in various intermolecular interactions. The found supramolecular motifs formed by the stronger hydrogen bonds are presented in Figure 7. To ease the analysis of supramolecular synthons the graph set notation was used [63,64].

In the case of 1-Ala, the supramolecular primary level motif is a C(4) chain formed between amide groups (E_tot = −55.6 kJ/mol), which is assisted by dispersion interactions between parallelly arranged amphiphilic molecules. However, it is not the strongest interaction in this crystal. The carboxylic acid fragments preserve the usual cyclic R^2^_2_(8) motif resulting in high total energy of ca. −116 kJ/mol. At the higher level of the graph set theory both of the mentioned motifs link into a R^4^_4_(24) ring. The final patterning in 1-Ala may be considered to be parallel tapes between which dispersion forces are present. Such arrangement does not seem to ease a possible formation of gel, since the hydrophilic parts are strongly bonded and the rearrangement of the molecules to accommodate solvent would demand a large energy input.

2-Pro exhibits only a simple C(7) herring-bone pattern via O–H…O_amide contact (E_tot = −42 kJ/mol). The maximum total energy of pairwise interaction is smaller than in the other structures (−46 kJ/mol) and comes mainly from dispersion term. This may indicate possible formation of polymorphs. However, the blocked N atom limits the prospects of this compound to interact freely with solvent molecules.

Comparing the crystal packing of 3-Phe, 4-Leu, and 5-Val, the main noticeable feature is the common second level graph set R^4^_4_(20) ring with four donors and four hydrogen bond acceptors, in which the same functional groups are involved. These crystals are not isostructural, since the alkyl parts of each are arranged differently. However, some degree of similarity among these lamellar structures is observed, which is also in accordance with the energy frameworks.

The 3-Phe structure is dominated by two interactions of very similar total energies (−68.7 and −62.7 kJ/mol); however, their origin is entirely different. One of the interactions is a strong O–H…O_amide hydrogen bond with the highest contribution of coulombic factor (−87.1 kJ/mol). The other one is N–H…O_acid with moderate coulombic (−36.6 kJ/mol) and quite high dispersion (−60.3 kJ/mol) inputs.

The strongest interaction in 4-Leu comes from O–H…O_amide hydrogen bond with high coulombic contribution (−77 kJ/mol) has a total energy of −54.5 kJ/mol. However, another pairwise total energy nearly equally strong is an effect of a combination of a weaker N–H…O_acid hydrogen bond (E_Coul = −31.5 kJ/mol) supported by high dispersion term (−52.6 kJ/mol) associated with a parallel alignment of the alkyl chains.

Structure of 5-Val is stabilized by similar interactions, but the much higher coulombic energetic term of ca. −92 kJ/mol in the case of O–H…O_amide h-bond is a consequence of an additional contribution of an accompanying C–H…O_acid contact which closes the R^2^_2_(8) ring. Additionally, the amide N–H…O_acid interaction is assisted by a weaker C–H…O_amide one (motif R^2^_2_(11)) giving the contribution to the electrostatic energy.

## 4. Conclusions

The crystal structures of a series of *N*-dodecanoyl-L-amino acid derivatives have been successfully determined. Energy frameworks analysis was applied for the first time to study the gelling behavior of a series of low-molecular-weight gelators, suitable for the formation of both organo- and hydrogels. The presence of carboxylic O–H and amide N–H groups impacted the most the crystal net energetics giving high coulombic contributions to the total energy in each of the studied structures. Three of the analyzed crystals (3-Phe, 4-Leu, and 5-Val) had the same cyclic supramolecular synthon R^4^_4_(20), stabilized by hydrogen bonds. The different alignment of the aliphatic chains had a minor contribution to the total energy in the crystals, but the alkyl chains exhibited wide range of conformations from synclinal (5-Val) through anticlinal (3-Phe) up to antiperiplanar (4-Leu) ones. This information may be useful when predicting the tangling ability of the lipophilic parts of the gelator molecules.

Spontaneous formation of self-assembled fibrillary network (SAFiN), at least in the case of apolar solvents, seems to have its origin in two phenomena: (1) the hydrophilic parts of amphiphilic organogelators interact to each other influencing the stability of the gels as well as the energy necessary to initiate the gelling process; (2) the hydrophobic fibers may tangle up trapping in voids lipophilic solvent molecules. 

The combination of crystallographic studies and energy frameworks analysis may be a useful help in understanding the gelling behavior and in designing new low-molecular-weight organogelator (LMOG) materials. 

## Figures and Tables

**Table 1:** Intermolecular energies calculated by CrystalExplorer at CE-B3LYP/6-31G(d,p) level in kJ/mol scaled by factors 1.057, 0.74, 0.871, and 0.618 for coulombic, polarization, dispersion, and repulsion energy terms, respectively, according to Mackenzie et al. [37].

Crystal	Symmetry Code	R [Å]	E_Coul	E_pol	E_dis	E_rep	E_tot
1-Ala	−	14.43	6.13	−0.96	−6.53	0.00	−1.36
	x, y, z	6.36	−6.24	−2.15	−31.70	15.51	−24.57
	x, y, z	4.89	−37.73	−7.03	−40.07	29.23	−55.60
	−	20.79	0.00	0.00	−1.65	0.00	−1.65
	x, y, z	5.60	−8.88	−2.22	−61.84	21.57	−51.37
	−	20.53	−1.69	0.00	−9.49	0.00	−11.19
	−	14.50	−84.67	−19.91	−11.93	0.00	−116.50
	−	17.57	10.46	−1.04	−2.00	0.00	7.43
	−	17.09	−11.42	−2.00	−8.54	0.00	−21.95
2-Pro	−x, y + 1/2, −z	10.56	−7.40	−0.59	−40.94	16.50	−32.43
	x, y, z	6.66	−14.59	−3.70	−50.87	22.99	−46.16
	−x, y + 1/2, −z	15.22	−0.95	−0.07	−16.64	0.00	−17.66
	−x, y + 1/2, −z	8.95	−0.53	−0.22	−6.01	0.62	−6.14
	x, y, z	8.08	−0.63	−0.52	−15.77	6.74	−10.18
	x, y, z	10.47	−2.01	−0.30	−10.10	3.21	−9.19
	−x, y + 1/2, −z	9.09	−60.14	−10.58	−14.37	43.20	−41.90
	−x, y + 1/2, −z	11.89	−14.38	−3.63	−20.21	12.30	−25.91
3-Phe	x + 1/2, −y + 1/2, −z	11.37	−8.4	−2.8	−55.7	31.8	−39.8
	x, y, z	5.34	−36.6	−10.4	−60.3	58.7	−62.7
	−x, y + 1/2, −z + 1/2	8.17	−3.4	−0.6	−20.3	12.0	−14.3
	−x + 1/2, −y, z + 1/2	15.28	1.5	−1.1	−35.0	0.0	−29.7
	−x, y + 1/2, -z + 1/2	7.49	−87.1	−20.7	−32.9	109.1	−68.7
	x + 1/2, −y + 1/2, −z	18.32	−2.3	−0.5	−13.4	0.0	−14.5
	−x + 1/2, −y, z + 1/2	15.85	1.2	−1.5	−13.1	0.0	−11.3
4-Leu	x, y, z	5.33	−31.50	−6.59	−52.61	33.06	−57.63
	−x, y + 1/2, −z + 1/2	9.66	−77.06	−13.54	−14.46	50.55	−54.50
	−x, −y + 1/2, z + 1/2	14.83	−1.16	−0.74	−11.93	0.00	−13.84
	−x, −y + 1/2, z + 1/2	15.69	5.92	−0.81	−14.63	0.00	−9.53
	x, −y, −z	17.76	2.43	−0.07	−6.62	0.00	−4.26
	−x, y + 1/2, −z + 1/2	7.93	−4.76	−0.44	−24.56	10.44	−19.32
	x, −y, −z	18.54	0.21	0.00	−0.87	0.00	−0.66
	x, −y, −z	10.74	−3.81	−0.37	−25.96	8.96	−21.17
	x, −y, −z	14.16	−2.22	−1.04	−6.62	0.00	−9.88
	−x, y + 1/2, −z + 1/2	9.44	0.42	−0.07	−3.75	0.31	−3.09
	x, −y, −z	9.32	−6.02	−0.15	−33.19	14.09	−25.27
5-Val	x, y, z	5.68	−43.44	−8.44	−45.64	45.42	−52.10
	−x, y + 1/2, −z + 1/2	6.80	−6.87	−1.18	−39.63	16.93	−30.75
	x + 1/2, −y + 1/2, −z	17.32	0.11	−0.15	−10.19	0.00	−10.23
	−x, y + 1/2, −z + 1/2	5.73	−91.96	−15.69	−46.60	80.53	−73.72
	x + 1/2, −y + 1/2, −z	17.85	−3.91	−0.96	−13.85	0.00	−18.72
	−x + 1/2, −y, z + 1/2	14.41	3.81	−0.07	−18.81	0.00	−15.08
	−x + 1/2, −y, z + 1/2	17.81	−1.27	0.00	−3.48	0.00	−4.75

R—distance between centers of molecules.

## Supplementary Materials

The following supporting information can be downloaded at: https://www.mdpi.com/article/10.3390/ma16020702/s1, Table S1: Crystal data and structure refinement for the studied compounds; Table S2: Selected bond lengths and torsions in the analysed structures [Å,°]; Figures S1–S5H NMR (500 MHz) and ^13^C NMR (125 MHz) spectra in CDCl_3_ for 1-Ala, 2-Pro, 3-Phe, 4-Leu, and 5-Val, respectively.

## References

[1] Bajaj S., Singla D., Sakhuja N. (2012). Stability testing of pharmaceutical products. J. Appl. Pharm. Sci..

[2] Kommanaboyina B., Rhodes C.T. (1999). Trends in stability testing, with emphasis on stability during distribution and storage. Drug Dev. Ind. Pharm..

[3] Dormont F., Brusini R., Cailleau C., Reynaud F., Reynaud F., Peramo A., Gendron A., Mougin J., Gaudin F., Gaudin F. (2020). Squalene-based multidrug nanoparticles for improved mitigation of uncontrolled inflammation in rodents. Sci. Adv..

[4] Ahmad K., Rabbani G., Baig M.H., Lim J.H., Khan M.E., Lee E.J., Ashraf G.M., Choi I. (2017). Nanoparticle-based drugs: A potential armamentarium of effective anti-cancer therapies. Curr. Drug Metab..

[5] Sharma A.K., Keservani R.K. (2019). Nanoconjugate Nanocarriers for Drug Delivery.

[6] McNeil S.E. (2011). Characterization of Nanoparticles Intended for Drug Delivery.

[7] Maeda H., Wu J., Sawa T., Matsumura Y., Hori K. (2000). Tumor vascular permeability and the EPR effect in macromolecular therapeutics: A review. J. Control Release.

[8] Sahoo S.K., Labhasetwar V. (2003). Nanotech approaches to drug delivery and imaging. Drug Discov. Today.

[9] Efentakis M., Pagoni I., Vlachou M., Avgoustakis K. (2007). Dimensional changes, gel layer evolution and drug release studies in hydrophilic matrices loaded with drugs of different solubility. Int. J. Pharm..

[10] Milton Harris J., Chess R.B. (2003). Effect of pegylation on pharmaceuticals. Nat. Rev. Drug Discov..

[11] Jani P., Halbert G.W., Langridge J., Florence A.T. (1990). Nanoparticle uptake by the rat gastrointestinal mucosa: Quantitation and particle size dependency. J. Pharm. Pharmacol..

[12] Jani P., Halbert G.W., Langridge J., Florence A.T. (1989). The uptake and translocation of latex nanospheres and microspheres after oral administration to rats. J. Pharm. Pharmacol..

[13] Gupta R.B., Kompella U.B. (2006). Nanoparticle Technology for Drug Delivery.

[14] Shapero K., Fenaroli F., Lynch I., Cottell D.C., Salvati A., Dawson K.A. (2011). Time and space resolved uptake study of silica nanoparticles by human cells. Mol. Biosyst..

[15] Schmid G. (2010). Nanoparticles: From Theory to Application: Second Edition.

[16] Turkevich J., Kim G. (1970). Palladium: Preparation and catalytic properties of particles of uniform size. Science.

[17] Reetz M.T., Maase M. (1999). Redox-controlled size-selective fabrication of nanostructured transition metal colloids. Adv. Mater..

[18] Tao A.R., Habas S., Yang P. (2008). Shape control of colloidal metal nanocrystals. Small.

[19] Poinern G.E.J. (2014). Laboratory Course in Nanoscience and Nanotechnology.

[20] McClements D.J. (2014). Nanoparticle- and Microparticle-Based Delivery Systems.

[21] Israelachvili J. (2011). Intermolecular and Surface Forces.

[22] Bastiat G., Leroux J.C. (2009). Pharmaceutical organogels prepared from aromatic amino acid derivatives. J. Mater. Chem..

[23] Gao Z.H., Crowley W.R., Shukla A.J., Johnson J.R., Reger J.F. (1995). Controlled release of contraceptive steroids from biodegradable and injectable gel formulations: In vivo evaluation. Pharm. Res..

[24] Vintiloiu A., Leroux J.C. (2008). Organogels and their use in drug delivery—A review. J. Control Release.

[25] Bielejewski M., Łapiński A., Demchuk O. (2017). Molecular interactions in high conductive gel electrolytes based on low molecular weight gelator. J. Colloid Interface Sci..

[26] Bielejewski M., Kowalczuk J., Kaszyńska J., Łapiński A., Luboradzki R., Demchuk O., Tritt-Goc J. (2013). Novel supramolecular organogels based on a hydrazide derivative: Non-polar solvent-assisted self-assembly, selective gelation properties, nanostructure, solvent dynamics. Soft Matter.

[27] Bhattacharya S., Krishnan-Ghosh Y. (2001). First report of phase selective gelation of oil from oil/water mixtures. Possible implications toward containing oil spills. Chem. Commun..

[28] Dasmandal S., Kundu A., Rudra S., Mahapatra A. (2015). Binding interaction of an anionic amino acid surfactant with bovine serum albumin: Physicochemical and spectroscopic investigations combined with molecular docking study. RSC Adv..

[29] Pal A., Ghosh Y.K., Bhattacharya S. (2007). Molecular mechanism of physical gelation of hydrocarbons by fatty acid amides of natural amino acids. Tetrahedron.

[30] Chen J., Boott C.E., Lewis L., Siu A., Al-Debasi R., Carta V., Fogh A.A., Kurek D.Z., Wang L., Maclachlan M.J. (2020). Amino acid-containing phase-selective organogelators: A water-based delivery system for oil spill treatment. ACS Omega.

[31] Oda R. (2006). Safin gels with amphiphilic molecules. Molecular Gels: Materials with Self-Assembled Fibrillar Networks.

[32] Dastidar P. (2008). Supramolecular gelling agents: Can they be designed?. Chem. Soc. Rev..

[33] Datta S., Bhattacharya S. (2015). Multifarious facets of sugar-derived molecular gels: Molecular features, mechanisms of self-assembly and emerging applications. Chem. Soc. Rev..

[34] Lombardo D., Kiselev M.A., Magazù S., Calandra P. (2015). Amphiphiles self-assembly: Basic concepts and future perspectives of supramolecular approaches. Adv. Condens. Matter Phys..

[35] Abdellatif M.M., Ibrahim S., Nomura K. (2020). Efficient and eco-friendly low-molecular-weight gelators based on l-phenylalanine as promising remediation tool for oil pollution. J. King Saud Univ. -Sci..

[36] Du X., Zhou J., Shi J., Xu B. (2015). Supramolecular hydrogelators and hydrogels: From soft matter to molecular biomaterials. Chem. Rev..

[37] Mongkholkeaw S., Songsasen A., Duangthongyou T., Chainok K., Suramitr S., Wattanathana W., Wannalerse B. (2020). Crystal structure, Hirshfeld surface analysis and computational study of 2-chloro-N-[4-(methylsulfanyl)phenyl]acetamide. Acta Crystallogr. Sect. E Crystallogr. Commun..

[38] Bojarska J., Remko M., Madura I.D., Wojciechowski J.M., Olczak A., Kaczmarek K., Zabrocki J., Wolf W.M. (2019). Supramolecular synthon polymorphism in modified amino acids. Structural, conformational and energy landscapes of N-benzoyl-2′-hydroxy-3-methylisovaline. J. Mol. Struct..

[39] Suresh M., Srinivasan K. (2022). Crystal structure and Hirshfeld surface analysis of dl-methionine polymorphs (α and β). J. Mol. Struct..

[40] Pierri G., Corno M., Macedi E., Voccia M., Tedesco C. (2021). Solid-state conformational flexibility at work: Energetic landscape of a single crystal-to-single crystal transformation in a cyclic hexapeptoid. Cryst. Growth Des..

[41] Thomas S.P., Spackman P.R., Jayatilaka D., Spackman M.A. (2018). accurate lattice energies for molecular crystals from experimental crystal structures. J. Chem. Theory Comput..

[42] Dey D., Bhandary S., Thomas S.P., Spackman M.A., Chopra D. (2016). Energy frameworks and a topological analysis of the supramolecular features in in situ cryocrystallized liquids: Tuning the weak interaction landscape via fluorination. Phys. Chem. Chem. Phys..

[43] Torubaev Y.V., Rai D.K., Skabitsky I.V., Pakhira S., Dmitrienko A. (2019). Energy framework approach to the supramolecular reactions: Interplay of the secondary bonding interaction in Ph2E2 (E = Se, Te)/p-I-C6F4-I co-crystals. New J. Chem..

[44] Poirier A., Le Griel P., Zinn T., Pernot P., Roelants S.L.K.W., Soetaert W., Baccile N. (2022). Energy landscape of the sugar conformation controls the sol-to-gel transition in self-assembled bola glycolipid hydrogels. Chem. Mater..

[45] Groom C.R., Bruno I.J., Lightfoot M.P., Ward S.C. (2016). The Cambridge structural database. Acta Crystallogr. Sect. B Struct. Sci. Cryst. Eng. Mater..

[46] Schade B., Fuhrhop J.H., Hubert V., Weber M., Luger P. (1997). Hydrogen-Bonding Networks Involving Water in Amphiphilic N-Dodecanoyl-l-serine Monohydrate. Acta Crystallogr. Sect. C Cryst. Struct. Commun..

[47] Ogawa T., Ohta A., Hata Y., Miyagishi S., Kunimoto K.K. (2006). (R)-1-phenylethylammonium N-tetradecanoyl-L-phenylalaninate monohydrate. Acta Crystallogr. Sect. E Struct. Rep. Online.

[48] Borkowski M., Orvalho S., Warszyński P., Demchuk O.M., Jarek E., Zawala J. (2022). Experimental and theoretical study of adsorption of synthesized amino acid core derived surfactants at an air/water interface. Phys. Chem. Chem. Phys..

[49] Hubalek F., Foger F.A. Fatty Acid Acylated D-Amino Acids for Oral Peptide Delivery, Patent no WO2014060447A1—World International Patent Organization 2014. https://patents.google.com/patent/WO2014060447A1/en.

[50] Sheldrick G.M. (2008). IUCr A short history of *SHELX*. Acta Crystallogr. Sect. A Found. Crystallogr..

[51] Dolomanov O.V., Bourhis L.J., Gildea R.J., Howard J.A.K., Puschmann H. (2009). OLEX2: A complete structure solution, refinement and analysis program. J. Appl. Crystallogr..

[52] Spackman M.A., Jayatilaka D. (2009). Hirshfeld surface analysis. CrystEngComm.

[53] Sahoo P.R., Kumar A., Kumar A., Kumar S. (2020). Experimental and computational investigation of polymorphism in methyl 3-hydroxy-4-(piperidin-1-ylmethyl)-2-naphthoate. J. Mol. Struct..

[54] Miroslaw B., Cristóvão B., Hnatejko Z. (2019). Halogen bonded lamellar motifs in crystals of Schiff base ZnII–LnIII–ZnII coordination compounds—Synthesis, structure, Hirshfeld surface analysis and physicochemical properties. Polyhedron.

[55] Mahmoudi G., Bauzá A., Rodríguez-Diéguez A., Garczarek P., Kaminsky W., Frontera A. (2015). Synthesis, X-ray characterization, DFT calculations and Hirshfeld surface analysis studies of carbohydrazone based on Zn(II) complexes. CrystEngComm.

[56] Spackman P.R., Turner M.J., McKinnon J.J., Wolff S.K., Grimwood D.J., Jayatilaka D., Spackman M.A. (2021). CrystalExplorer: A program for Hirshfeld surface analysis, visualization and quantitative analysis of molecular crystals. J. Appl. Crystallogr..

[57] Tan S.L., Jotani M.M., Tiekink E.R.T. (2019). Utilizing Hirshfeld surface calculations, non-covalent inter action (NCI) plots and the calculation of inter action energies in the analysis of mol ecular packing. Acta Crystallogr. Sect. E Crystallogr. Commun..

[58] Mackenzie C.F., Spackman P.R., Jayatilaka D., Spackman M.A. (2017). CrystalExplorer model energies and energy frame-works: Extension to metal coordination compounds, organic salts, solvates and open-shell systems. IUCrJ.

[59] Frisch M.J., Trucks G.W., Schlegel H.B., Scuseria G.E., Robb M.A., Cheeseman J.R., Scalmani G., Barone V., Petersson G.A., Nakatsuji H. (2016). Gaussian 09, Revision A.02.

[60] Johansson S.J.R., Johannessen T., Ellefsen C.F., Ristun M.S., Antonsen S., Hansen T.V., Stenstrom Y., Nolsoe J.M.J. (2019). A convenient protocol for the synthesis of fatty acid amides. Synlett.

[61] Kalek M., Fu G.C. (2015). Phosphine-catalyzed doubly stereoconvergent γ-additions of racemic heterocycles to racemic allenoates: The catalytic enantioselective synthesis of protected α,α-disubstituted α-amino acid derivatives. J. Am. Chem. Soc..

[62] Kowalczuk J., Łapiński A., Stolarczyk E., Demchuk O.M., Kubiński K., Janeczko M., Martyna A., Masłyk M., Turczyniak-Surdacka S. (2021). New Supramolecular Drug Carriers: The Study of Organogel Conjugated Gold Nanoparticles. Molecules.

[63] Etter M.C., MacDonald J.C., Bernstein J. (1990). Graph-set analysis of hydrogen-bond patterns in organic crystals. Acta Crystallogr. Sect. B Struct. Sci..

[64] Grell J., Bernstein J., Tinhofer G. (1999). Graph-set analysis of hydrogen-bond patterns: Some mathematical concepts. Acta Crystallogr. Sect. B Struct. Sci..

