# Supplementary material for: Low-Molecular-Weight Organogelators Based on N-dodecanoyl-L-amino Acids—Energy Frameworks and Supramolecular Synthons
Source: Materials (Basel). 2023 Jan 11;16(2):702. doi: 10.3390/ma16020702 (PMC9867098; doi:10.3390/ma16020702)
Supplement: Supplementary file 1 [file materials-16-00702-s001.zip › materials-2132875-supplementary.pdf]

Article

Low-molecular-weight organogelators based on *N*-dodecanoyl-L-amino acids – energy frameworks and supramolecular synthons

Barbara Miroslaw [1],*, Oleg M. Demchuk [2], Roman Luboradzki [3] and Katarzyna Tyszczuk-Rotko [1]

[1] Institute of Chemical Sciences, Faculty of Chemistry, Maria Curie-Sklodowska University in Lublin, 20-031 Lublin, Poland
[2] Faculty of Medicine, The John Paul II Catholic University of Lublin, 1h-Konstantynów St., 20-708 Lublin, Poland
[3] Institute of Physical Chemistry, Polish Academy of Sciences, 01-224 Warsaw, Poland
* Correspondence: barbara.miroslaw@mail.umcs.pl

Table S1 Crystal data and structure refinement for the studied compounds.
Table S2 Selected bond lengths and torsions in the analysed structures [Å,°].
Figure S1. ^{1}H NMR (500 MHz) and ^{13}C NMR (125 MHz) spectra in $CDCl_3$ for 1-Ala.
Figure S2. ^{1}H NMR (500 MHz) and ^{13}C NMR (125 MHz) spectra in $CDCl_3$ for 2-Pro.
Figure S3. ^{1}H NMR (500 MHz) and ^{13}C NMR (125 MHz) spectra in $CDCl_3$ for 3-Phe.
Figure S4. ^{1}H NMR (500 MHz) and ^{13}C NMR (125 MHz) spectra in $CDCl_3$ for 4-Leu.
Figure S5. ^{1}H NMR (500 MHz) and ^{13}C NMR (125 MHz) spectra in $CDCl_3$ for 5-Val.

Table S1 Crystal data and structure refinement for the studied compounds.

Identification code	**1-Ala**	**2-Pro**	**3-Phe**	**4-Leu**	**5-Val**
Empirical formula	$C_{15}H_{29}NO_3$	$C_{17}H_{31}NO_3$	$C_{21}H_{33}NO_3$	$C_{18}H_{35}NO_3$	$C_{17}H_{33}NO_3$
Formula weight	271.39	297.43	347.48	313.47	299.44
Temperature/K	120.10(10)	120.10(10)	120.10(10)	120.10(10)	120.10(10)
Crystal system	triclinic	monoclinic	orthorhombic	orthorhombic	orthorhombic
Space group	P1	$P2_1$	$P2_12_12_1$	$P22_12_1$	$P2_12_12_1$
a/Å	4.8852(2)	6.65959(13)	5.3354(2)	5.32892(18)	32.7663(7)
b/Å	5.5958(2)	8.08116(17)	12.7541(6)	13.4149(4)	9.9958(2)
c/Å	30.2891(13)	15.9905(3)	28.9824(14)	27.0501(8)	5.68440(11)
α/°	90.765(3)	90	90	90	90
β/°	91.034(4)	90.4021(17)	90	90	90
γ/°	105.561(4)	90	90	90	90
Volume/$Å^3$	797.37(6)	860.54(3)	1972.19(16)	1933.73(10)	1861.79(7)
Z	2	2	4	4	4
$\rho_{calc}g/cm^3$	1.1303	1.1478	1.1703	1.0767	1.0677
μ/mm^{-1}	0.615	0.612	0.607	0.564	0.566
F(000)	300.9	329.0	762.3	698.0	664.0
Crystal size/mm^3	0.35 × 0.20 × 0.15	0.37 × 0.20 × 0.18	0.36 × 0.18 × 0.18	0.40 × 0.20 × 0.18	0.40 × 0.22 × 0.18
Radiation	CuKα (λ = 1.54184)	CuKα (λ = 1.54184)	CuKα (λ = 1.54184)	CuKα (λ = 1.54184)	CuKα (λ = 1.54184)
2Θ range for data collection/°	5.84 to 144.06	5.52 to 143.54	6.1 to 140.02	6.54 to 144.2	9.24 to 143.98
Index ranges	-4 ≤ h ≤ 5, -6 ≤ k ≤ 6, -37 ≤ l ≤ 37	-6 ≤ h ≤ 8, -9 ≤ k ≤ 6, -19 ≤ l ≤ 18	-6 ≤ h ≤ 5, -15 ≤ k ≤ 14, -31 ≤ l ≤ 34	-6 ≤ h ≤ 4, -16 ≤ k ≤ 16, -29 ≤ l ≤ 32	-39 ≤ h ≤ 36, -12 ≤ k ≤ 10, -6 ≤ l ≤ 6
Reflections collected	15118	4825	5142	6310	8144
Independent reflections	5297 [R_{int} = 0.0340, R_{sigma} = 0.0330]	2282 [R_{int} = 0.0146, R_{sigma} = 0.0165]	3303 [R_{int} = 0.0432, R_{sigma} = 0.0541]	3157 [R_{int} = 0.0240, R_{sigma} = 0.0220]	3259 [R_{int} = 0.0275, R_{sigma} = 0.0228]
Data/restraints/parameters	5297/0/354	2282/0/191	3303/0/227	3157/0/303	3259/0/204
Goodness-of-fit on F^2	1.218	0.868	1.092	1.067	1.786
Final R indexes [I>=2σ (I)]	R_1 = 0.0448	R_1 = 0.0297	R_1 = 0.0487	R_1 = 0.0387	R_1 = 0.0639
Final R indexes [all data]	R_1 = 0.0472, wR_2 = 0.1537	R_1 = 0.0306, wR_2 = 0.0900	R_1 = 0.0524, wR_2 = 0.1494	R_1 = 0.0393, wR_2 = 0.1046	R_1 = 0.0662, wR_2 = 0.2052
Largest diff. peak/hole / e $Å^{-3}$	0.32/-0.26	0.18/-0.22	0.24/-0.24	0.19/-0.21	0.50/-0.42
Flack parameter	0.18(18)	0.03(13)	0.1(3)	0.00(18)	-0.1(3)
CCDC No.	2226607	2226609	2231565	2226608	2226610
			Redetermination of structure [1]		

1. Chen, J.; Boott, C.E.; Lewis, L.; Siu, A.; Al-Debasi, R.; Carta, V.; Fogh, A.A.; Kurek, D.Z.; Wang, L.; Maclachlan, M.J.; et al. Amino Acid-Containing Phase-Selective Organogelators: A Water-Based Delivery System for Oil Spill Treatment. *ACS Omega* **2020**, *5*, 18758–18765. CCDC No. 1959468, CSD Refcode: WUWJUW.

Table S2 Selected bond lengths and torsions in the analysed structures [Å,°].

Bond	**1-Ala**	**2-Pro**	**3-Phe**	**4-Leu**	**5-Val**
O1-C1	1.280(3)	1.3260(15)	1.313(3)	1.315(2)	1.312(3)
O2-C1	1.244(3)	1.2040(15)	1.217(3)	1.211(2)	1.219(3)
O3-C3	1.225(3)	1.2473(15)	1.246(3)	1.242(2)	1.253(3)
N1-C2	1.462(2)	1.4561(16)	1.458(3)	1.453(2)	1.457(3)
N1-C3	1.339(3)	1.3404(16)	1.336(3)	1.343(2)	1.336(3)
O4-C16	1.278(3)				
O5-C16	1.259(3)				
O6-C18	1.228(3)				
N2-C17	1.448(2)				
N2-C18	1.352(2)				
Torsion					
N1-C3-C4-C5	143.7(2)	159.7(1)	-119.3(2)	-125.4(2)	-80.5(3)
N2-C18-C19-C20	-163.5(2)				
C1-C2-N1-C3	55.0(3)	-65.3(1)	-102.6(2)	-110.1(2)	-70.9(3)
C16-C17-N2-C18	-76.8(2)				
C3-C4-C5-C6	-175.1(2)	-63.5(1)	-172.1(2)	179.1(1)	-55.8(3)
C18-C19-C20-C21	-177.6(2)				

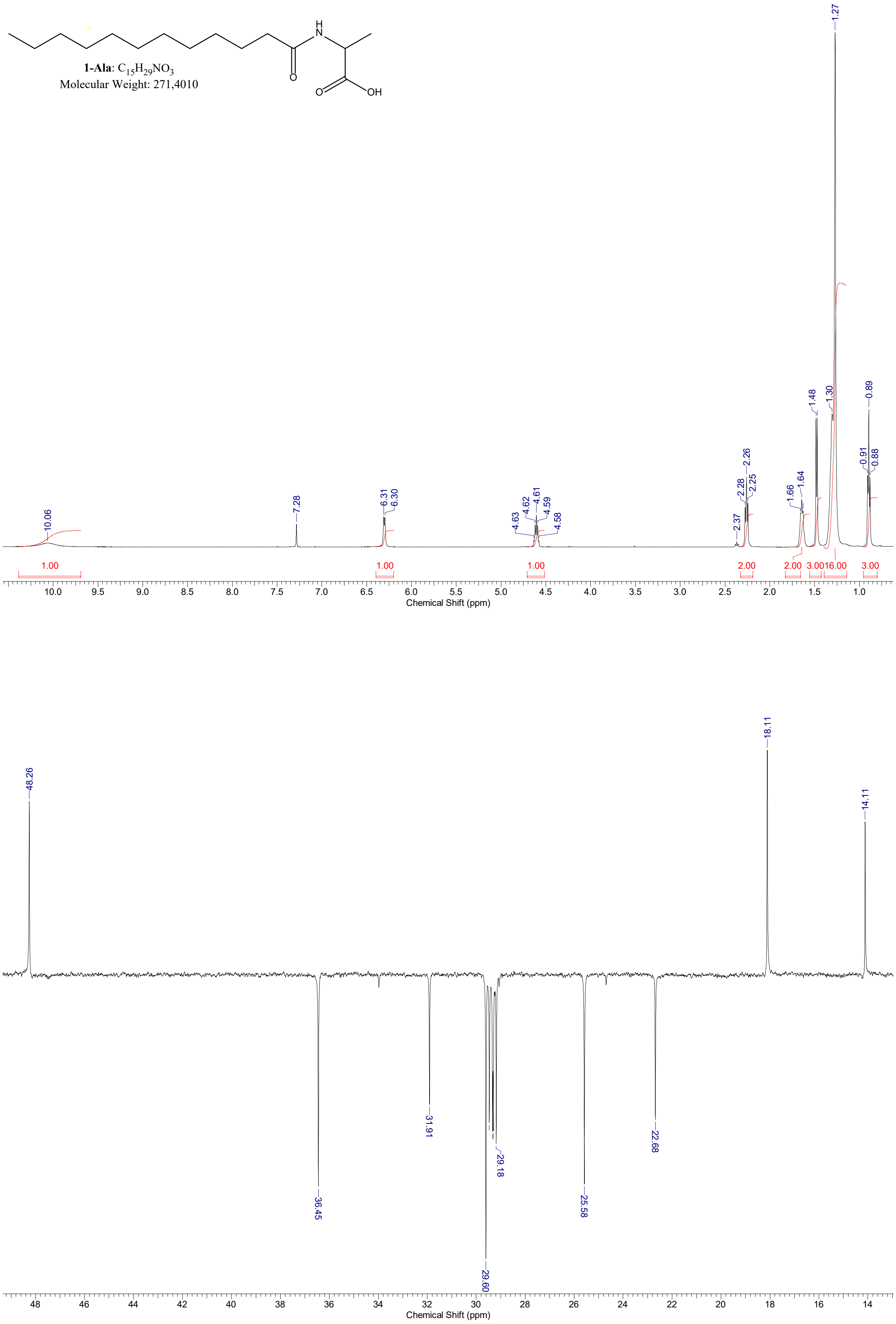

Figure S1. 1H NMR (500 MHz) and 13C NMR (125 MHz) spectra in CDCl3 for 1-Ala.

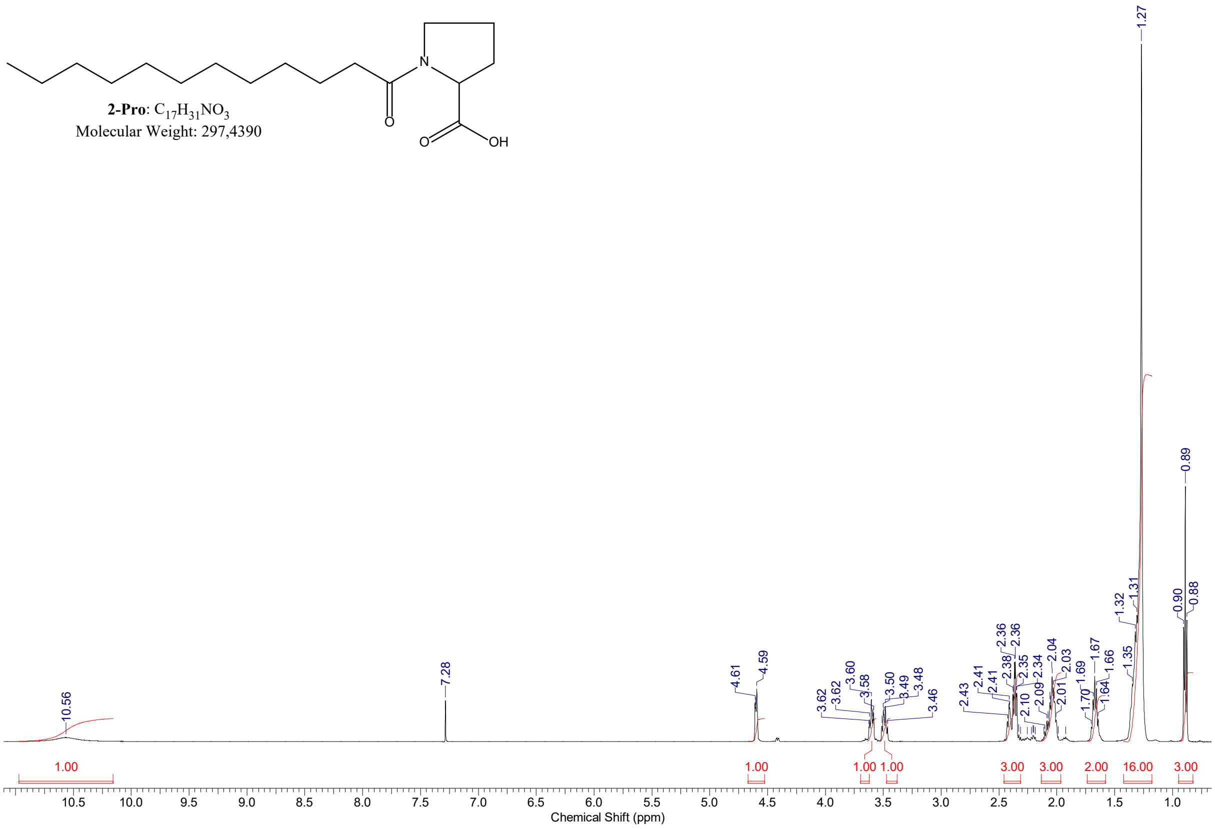

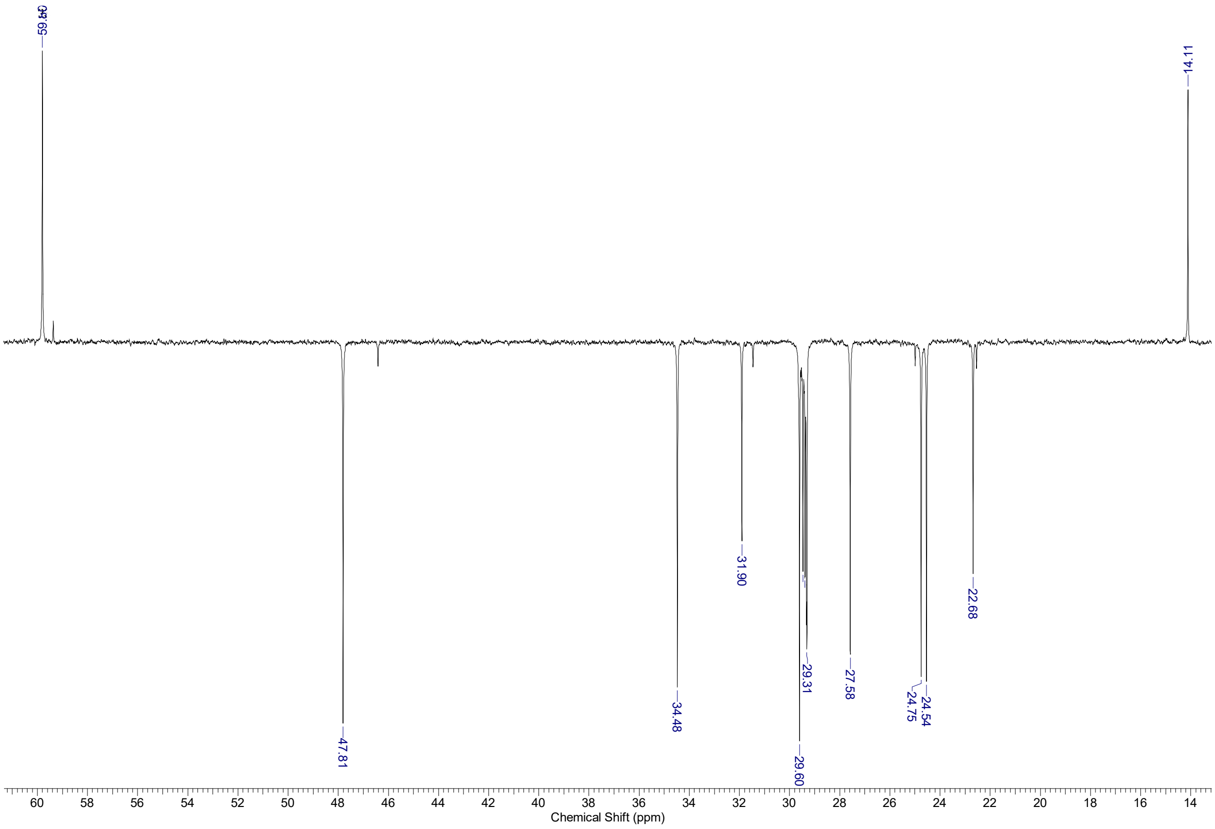

Figure S2. 1H NMR (500 MHz) and 13C NMR (125 MHz) spectra in CDCl3 for 2-Pro.

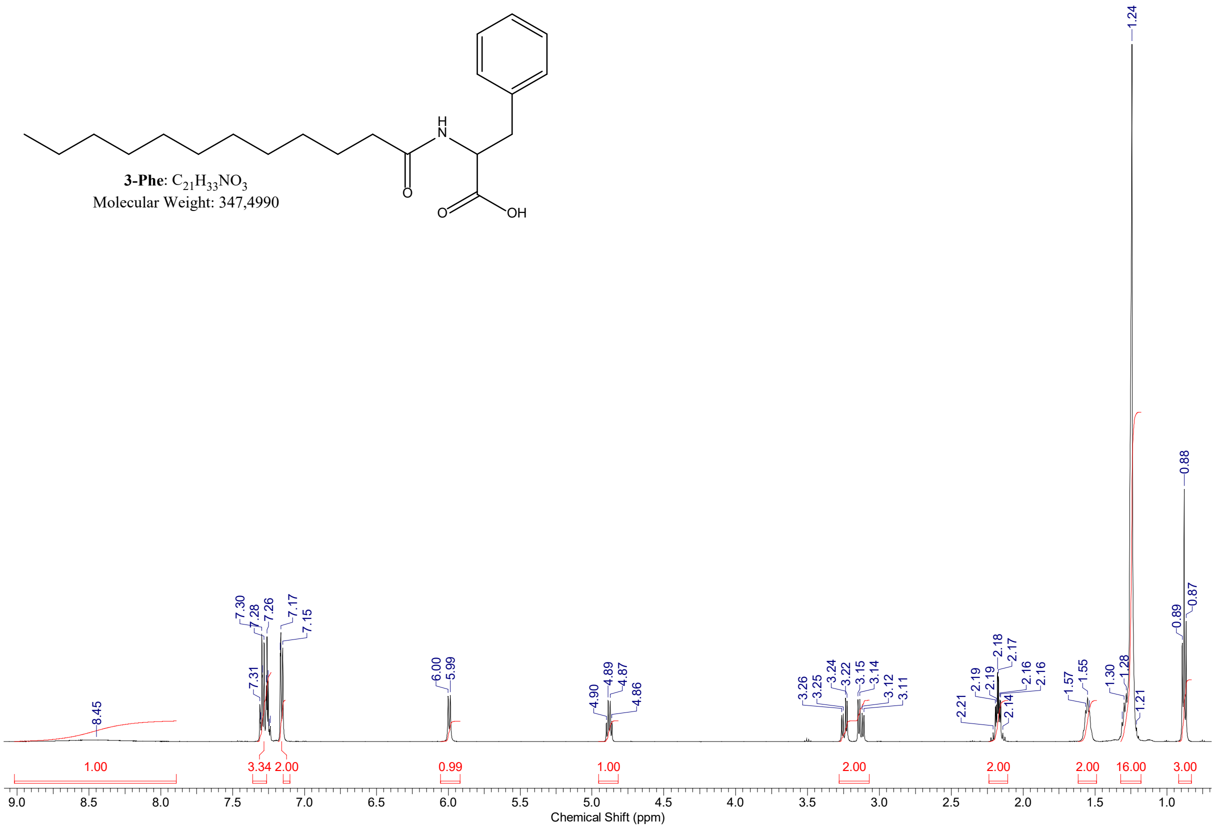

3-Phe: $C_{21}H_{33}NO_3$
Molecular Weight: 347,4990

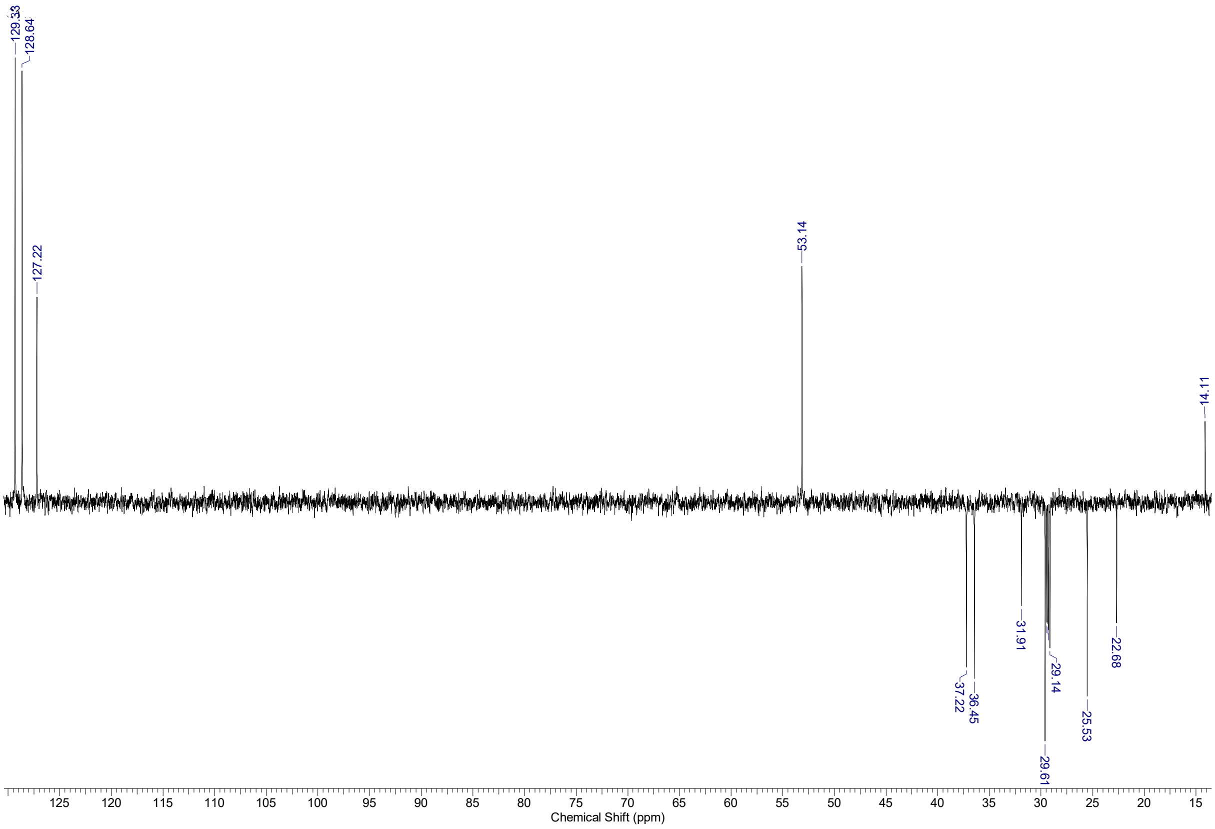

Figure S3. 1H NMR (500 MHz) and 13C NMR (125 MHz) spectra in CDCl3 for 3-Phe.

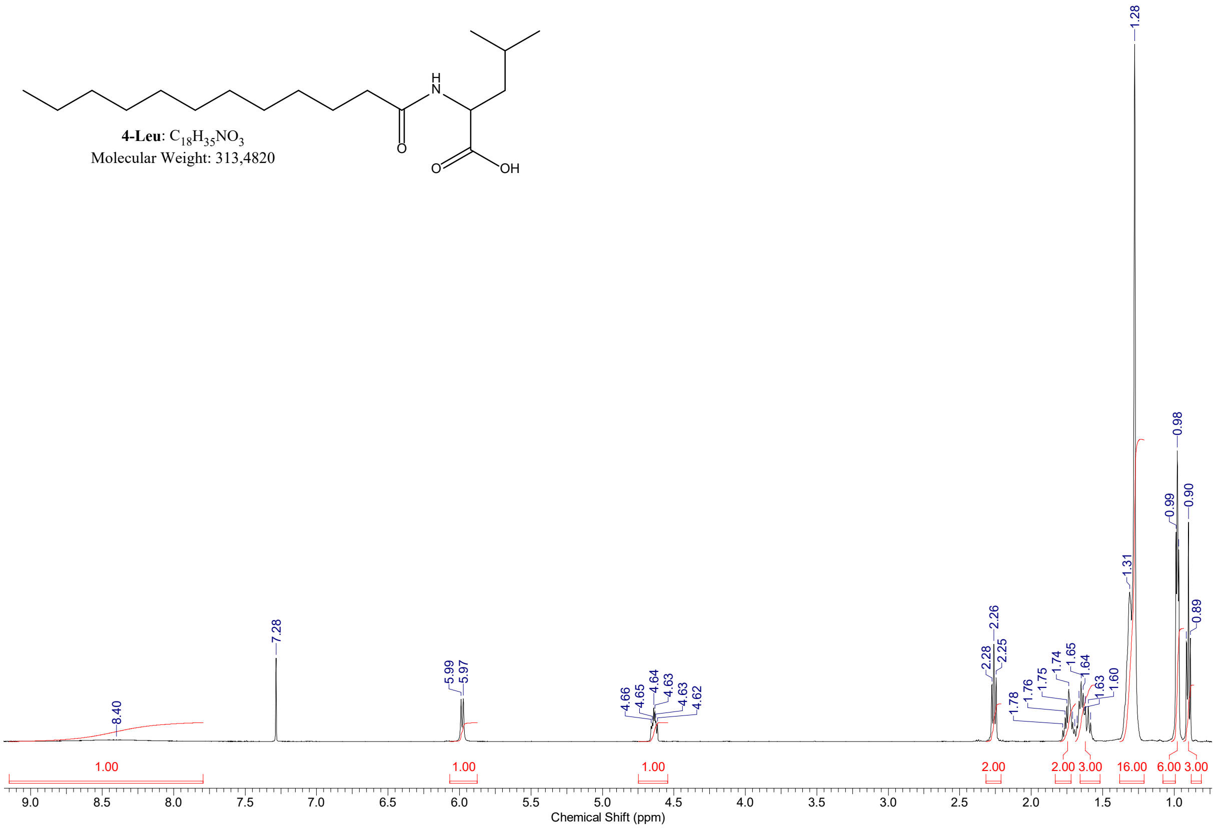

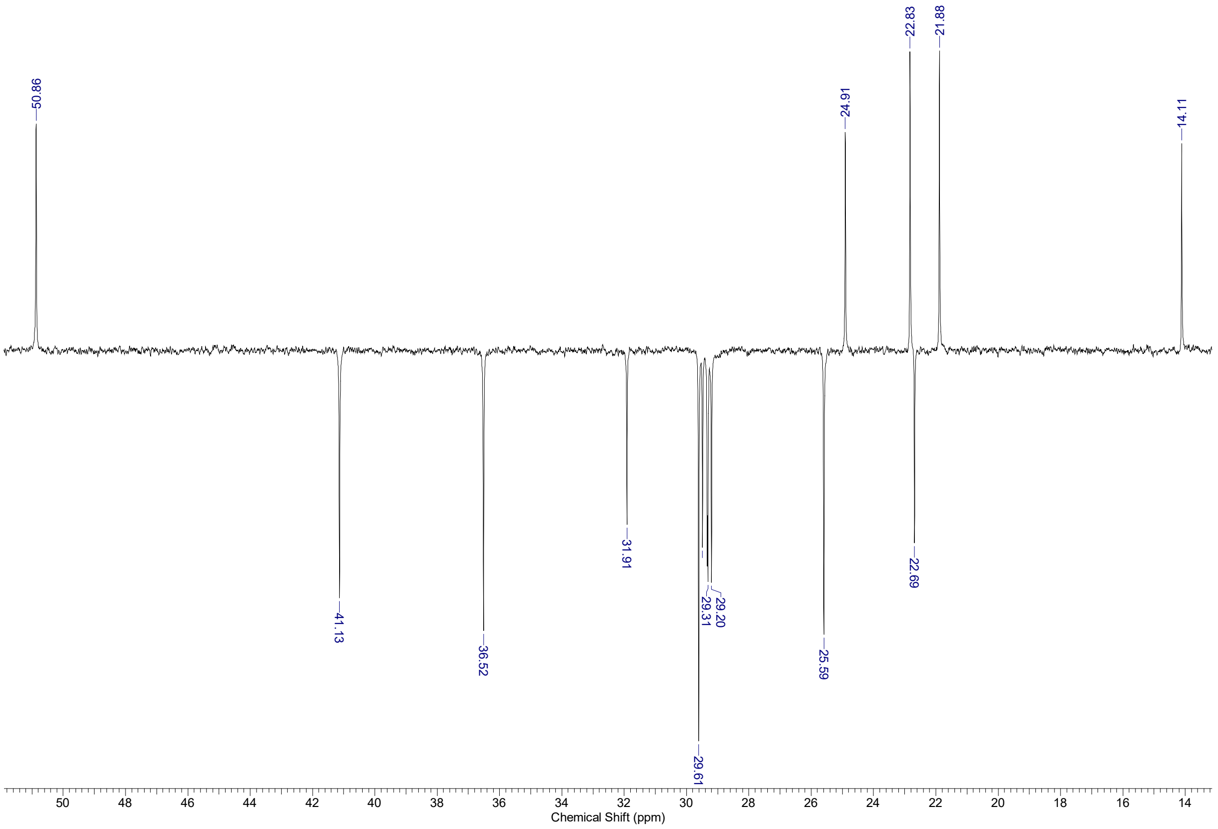

Figure S4. 1H NMR (500 MHz) and 13C NMR (125 MHz) spectra in CDCl3 for 4-Leu.

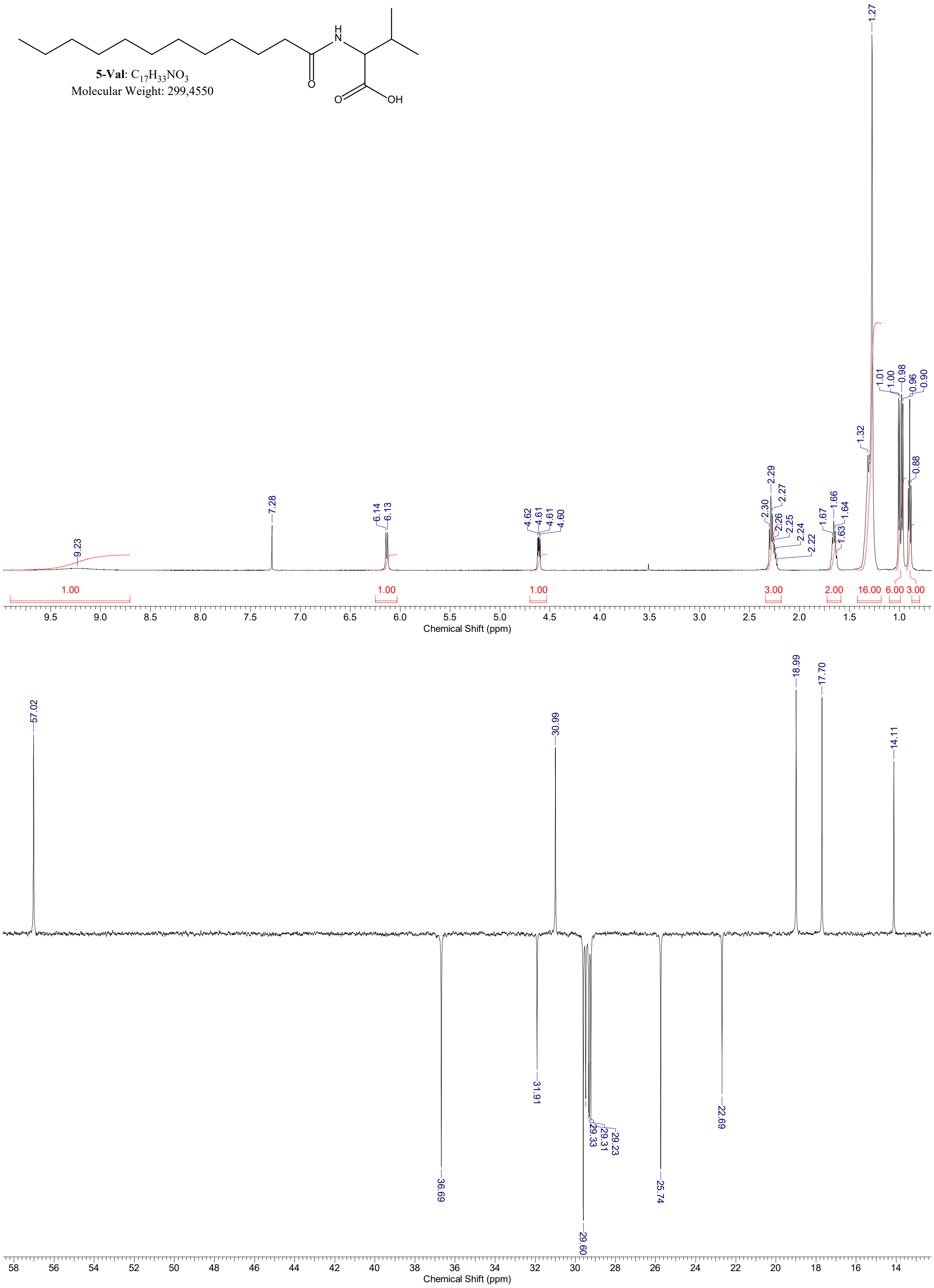

Figure S5. 1H NMR (500 MHz) and 13C NMR (125 MHz) spectra in CDCl3 for 5-Val.
